# Deworming in pre-school age children: A global empirical analysis of health outcomes

**DOI:** 10.1371/journal.pntd.0006500

**Published:** 2018-05-31

**Authors:** Nathan C. Lo, Jedidiah Snyder, David G. Addiss, Sam Heft-Neal, Jason R. Andrews, Eran Bendavid

**Affiliations:** 1 Division of Epidemiology, Stanford University School of Medicine, Stanford, CA, United States of America; 2 Division of Infectious Diseases and Geographic Medicine, Stanford University School of Medicine, Stanford, CA, United States of America; 3 Children Without Worms, Task Force for Global Health, Decatur, GA, United States of America; 4 Rollins School of Public Health, Emory University, Atlanta, GA, United States of America; 5 Department of Earth System Science, Stanford University, Stanford, CA, United States of America; 6 Primary Care and Population Health, Stanford University, Stanford, CA, United States of America; 7 Center for Health Policy and the Center for Primary Care and Outcomes Research, Stanford University, Stanford, CA, United States of America; McGill University, CANADA

## Abstract

**Background:**

There is debate over the effectiveness of deworming children against soil-transmitted helminthiasis (STH) to improve health outcomes, and current evidence may be limited in study design and generalizability. However, programmatic deworming continues throughout low and middle-income countries.

**Methodology and principal findings:**

We performed an empirical evaluation of the relationship between deworming in pre-school age children (ages 1–4 years) within the previous 6 months, as proxy-reported by the mother, and health outcomes of weight, height, and hemoglobin. We used nationally representative cross-sectional data from 45 countries using the Demographic and Health Surveys (DHS) during the period 2005–2016. We used logistic regression with coarsened exact matching, fixed effects for survey and year, and person-level covariates. We included data on 325,115 children in 45 STH-endemic countries from 66 DHS surveys. Globally in STH-endemic countries, children who received deworming treatment were less likely to be stunted (1.2 percentage point decline from mean of 36%; 95% CI [-1.9, -0.5%]; p<0.001), but we did not detect consistent associations between deworming and anemia or weight. In sub-Saharan Africa, we found that children who received deworming treatment were less likely to be stunted (1.1 percentage point decline from mean of 36%; 95% CI [-2.1, -0.2%]; p = 0.01) and less likely to have anemia (1.8 percentage point decline from mean of 58%; 95% CI [-2.8, -0.7%]; p<0.001), but we did not detect consistent associations between deworming and weight. These findings were robust across multiple statistical models, and we did not find consistently measurable associations with data from non-endemic settings.

**Conclusions and significance:**

Among pre-school age children, we detected a robust and consistent association between deworming and reduced stunting, with additional evidence for reduced anemia in sub-Saharan Africa. We did not find a consistent relationship between deworming and improved weight. This global empirical analysis provides evidence to support the deworming of pre-school age children.

## Author summary

Soil-transmitted helminths are parasitic worms that affect 1.5 billion people. The global public health strategy is regular deworming of children in endemic settings. In this study, we bring an independent dataset with over 320,000 pre-school age children across 45 STH-endemic countries to investigate the relationship between deworming and health outcomes of weight, height, and anemia. We address potential limitations of past studies by examining across diverse settings for improved generalizability, increasing sample size, and focusing on children who received treatment to improve statistical power. We find that deworming is robustly associated with reduced stunting in pre-school age children and find additional evidence that deworming may be associated with reduced anemia in sub-Saharan Africa. Deworming was not consistently associated with improved weight. These findings were robust across multiple statistical models, and we did not find consistently measurable associations with data from non-endemic settings, which improves confidence in the study findings. This global empirical analysis provides evidence to support the continued deworming of pre-school age children in endemic settings.

## Introduction

The large-scale empiric treatment of children for soil-transmitted helminthiasis (STH), commonly known as “deworming”, has become controversial [1–4]. This strategy of regular deworming (also known as ‘preventive chemotherapy’) with albendazole or mebendazole has been the main STH control strategy recommended by the World Health Organization (WHO) for over a decade [5]. While deworming continues to be implemented throughout low and middle-income countries for the estimated 1.5 billion people with STH (caused by infection with *Ascaris lumbricoides*, hookworm species of *Ancylostoma duodenale* and *Necator americanus*, and *Trichuris trichiura*), the evidence linking real-world experience of deworming with health outcomes is mixed [6].

Some randomized-controlled trials have demonstrated possible positive benefits of deworming on child health, primarily focused on weight or height, although many trials have not found these relations [3, 7–10]. Observational studies provide evidence for an association between deworming and cognitive improvements, physical growth, and reduced anemia [11–13]. However, many of these benefits have been disputed by recent studies [3, 4, 14, 15]. The Cochrane Collaboration (2015) and Campbell Collaboration (2017) systematic reviews and meta-analyses of randomized trials concluded deworming does not result in improved health outcomes, although they did find some benefit to treating those known to be infected [3, 4]. This was followed by substantial debate on the potential limitations of randomized trials and the Cochrane and Campbell meta-analyses [1, 2, 16–18]. In particular, because a minority of individuals harbors a majority of the worm burden, critics argued that these studies were underpowered to detect a meaningful effect, since only a fraction of the population benefits from treatment in areas of low or even moderate parasite burden. Furthermore, low STH prevalence in some clinical trials, heterogeneity in helminth species and their respective health effects, and the short time period of most trials may obscure potential health benefits. Uncertainty about the benefits of deworming is compounded by clustering of existing randomized trials in a few regions, which may limit generalizability to different contexts.

Periodic deworming of pre-school age children (ages 1–4 years) has been recommended by the WHO in areas where prevalence is ≥20% by stool microscopy since the 2006 WHO guidelines [5]. The strategy is intended to reduce the number of children with moderate and high-intensity infection, which is associated with adverse health outcomes [5]. Since pre-school age children (ages 1–4 years) are not typically reached through school-based deworming programs, they more commonly receive treatment through Child Health days [19], community-based deworming for lymphatic filariasis, or though the healthcare system. Recent modeling studies also have highlighted the importance of treating this population in order to reduce overall transmission and improve the cost-effectiveness of deworming programs [20–23]. From a measurement standpoint, this age group may enable more sensitive detection of health, educational, and economic benefits in response to deworming than the school-age population, which is more often studied. This is because periodic deworming in pre-school age children can occur before significant cumulative exposure to STH and continues throughout a period of rapid growth and development [10, 24].

To better understand the relationship between deworming and health in pre-school age children, we performed an empirical analysis using data from the Demographic and Health Surveys (DHS) across 45 countries. We addressed several limitations of past approaches in terms of generalizability, and draw upon a decade of deworming to measure population-level relationships between deworming and health.

## Methods

We examined the relationship between reported deworming and the pre-specified health outcomes of malnutrition (underweight, stunting) and anemia among pre-school age children (ages 1–4 years, selected based on WHO classification of this age group) in 45 low- and middle-income STH-endemic countries in Africa, the Americas, Asia, and Europe using cross-sectional data during the period 2005–2016. We also performed an analysis for sub-Saharan Africa alone given the substantial STH burden and scale of ongoing deworming [25].

### Data sources and study population

We used the DHS to examine the relationship between deworming exposure and health outcomes using cross-sectional data. These nationally representative surveys are conducted approximately every 5 years in many low- and middle-income countries in collaboration with in-country partners [26]. We used all available DHS surveys that included data on deworming exposure, health outcomes, and relevant covariates from endemic countries based on WHO classification (Appendix). DHS did not provide person-level information on STH infection status. We included all surveys from eligible countries in order to maximize sample size and examine temporal trends, and selected only children ages 1–4 years. We excluded countries with low-burden STH (defined as countries in which <10% children lived in areas where STH was endemic and WHO recommends mass deworming) or those missing data on deworming, health outcomes, or relevant covariates (see Appendix). A table of excluded surveys is available in the Appendix. We used the WHO preventive chemotherapy database to estimate country-level disease burden for STH based on proportion of children requiring deworming [27]. All data files are available from the DHS database online. This study relied on published data and did not require human subject research approval.

### Study measures and variables

We defined the study exposure using the mother’s report of whether her children received “drugs for intestinal parasites in the 6 months prior to the survey”. We examined three pre-specified binary health outcomes for pre-school age children: underweight, stunting, and anemia, which are the relevant health outcomes available in DHS surveys for this age group. We defined being underweight as a weight-for-age two standard deviations below the WHO global reference standard, stunting as a height-for-age two standard deviations below the WHO global reference standard, and anemia as a hemoglobin below 11 g/dL [28]. We chose to dichotomize health outcome variables in the main analysis to isolate the disease state and to account for possible confounding trends (e.g. increasing obesity), although we also conducted the analysis with continuous outcome variables. We included pre-specified person-level covariates in the analysis to address potential confounding relations, including child’s age and gender, mother’s age (<30 years or older based on prior DHS analysis on health-seeking behavior [29]) and education (defined as no education, some primary school, and completion of primary school or higher education), wealth quintile (a composite measure for each person’s household, relative based on country standard), place of residence (urban or rural, relative based on country’s definition), and binary variables indicating access to improved water, sanitation (based on toilet facility using the Joint Monitoring Programme definitions) [30], and healthcare-seeking behaviors using receipt of third dose of diphtheria-tetanus-pertussis vaccine as an indirect proxy (Appendix).

### Statistical analysis

The primary analyses used three health outcomes (underweight, stunting, or anemia) as dependent variables in logistic regression models where the primary independent variable was an indicator of receipt of deworming. We pre-processed our sample to improve balance between the treated and untreated groups using coarsened exact matching (CEM) within each DHS survey [31]. We used CEM over the more common propensity score matching to relax concerns over balance (i.e., CEM creates strata to ensure that treatment and untreated groups are matched on all covariates, rather than balanced on a propensity score alone), improve the intuitive understanding of the matching process, and because CEM was found to out-perform propensity score matching in balancing groups [32, 33] (see Appendix).

In all models we also used survey fixed effects, implemented as a set of indicator variables that control for all invariant differences between surveys (e.g. level of economic development and national STH prevalence at time of survey). Notably, these fixed effects control for unobserved sub-national (regional) differences in wealth and access to services or nutritional programs that would be related to health outcome, and are in addition to person-level covariates. We also included year fixed effects that control for common time effects across all surveys. Matched models using CEM do not include covariates in regression since they are pre-processed for balance. We estimated the odds ratios (OR) for a given health outcome among children that were dewormed relative to those that were not. We also reported the marginal effects, which represent the change in the probability of the outcome among those exposed to deworming relative to those that were not. We used robust standard errors clustered by country and survey. We also estimated the possible impact of providing deworming in the sub-Saharan African study countries by computing the number of avertable cases of disease states that could be related to deworming for better conceptualization of magnitude of the study results (see Appendix). The analytic code is available on request to the corresponding author.

### Sensitivity and other analyses

We conducted sensitivity analyses to examine the stability of our findings to alternative statistical models and assumptions on the data. We repeated the main analysis with alternative model specifications (e.g. ordinary least squares model with continuous outcomes), additional covariates (e.g. vitamin A receipt, use of malaria net, and breastfeeding), and country-specific analyses (see Appendix). We tested region (sub-national) fixed effects to match children within region. We defined relationship robustness in reference to these alternative statistical models.

We performed additional analyses including dose-response, negative controls, and sub-group analyses. The analysis tested stratification by age and estimated STH disease burden, including formal tests of interaction, to estimate dose response. We tested for the possibility of unobserved confounding by testing “negative controls” (factors theoretically unrelated to exposure and outcome, but possibly confounded by those same unobserved factors); we tested negative control exposures unrelated to deworming (e.g., “heard of family planning on radio” and “access to condoms”) and negative control outcomes (e.g., “cough in last two weeks” and “fever in last two weeks”) (see Appendix). We defined a relationship between deworming and health outcomes as “consistent” when the negative controls did not hold comparable associations to the primary study findings. We also examined the relationship between deworming and health outcomes in the sub-group of disease state (e.g. underweight children only). The Appendix contains additional details.

## Results

### Country descriptive analysis

**Fig 1:**
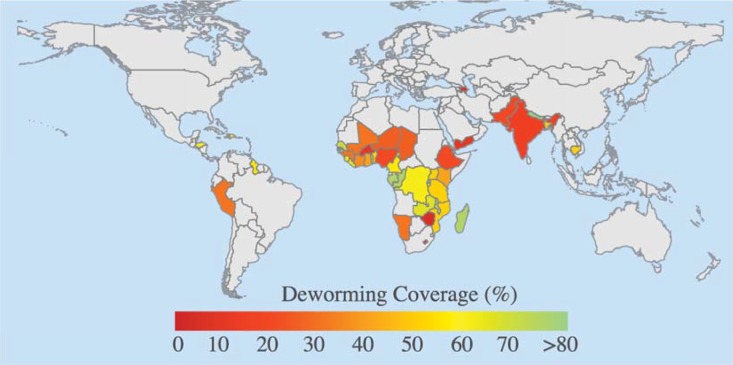
Study countries and deworming coverage over 2005–2016. We used data from the DHS for 45 STH endemic countries. For each, we computed the deworming coverage in pre-school age children.

This study included 325,115 pre-school age children from 45 countries in Africa, the Americas, Asia, and Europe from 2005 to 2016 (Table 1). This included a total of 66 DHS surveys, and excluded an additional 26 surveys for lack of data or endemicity for STH (see Appendix, Section 1). Over this time period, the mean proportion of respondents that indicated their pre-school age child received deworming medicine in the previous six months was 43.4%. The country-level deworming coverage was highly variable, ranging from 6.1% (Azerbaijan) to 87.4% (Rwanda), which may partly reflect the proportion of “at-risk” children within the country (Fig 1; Table A1). The overall prevalence of underweight, stunting, and anemia was 29.5%, 35.5%, and 53.0%, respectively. We found that children who received deworming were slightly older, wealthier, more likely to live in urban areas, had mothers that were more educated, had stronger health-seeking behavior, and greater access to toilets (Table 2). The CEM matched dewormed and untreated groups on all specified covariates (Table 2).

**Table 1:** Study countries and DHS survey year.

Study country	Survey years	Study country	Survey years
**Africa Region**		**Americas Region**	
Benin	2011–12	Dominican Republic	2007, 2013
Burkina Faso	2010	Guyana	2009
Burundi	2010	Haiti	2005–06, 2012
Cameroon	2011	Honduras	2005–06, 2011–12
Chad	2014–15	Peru	2005–08, 2012
Comoros	2012		
Côte d’Ivoire	2011–12	**South-East Asia Region**	
Democratic Republic of the Congo	2007, 2013–14	Bangladesh	2011, 2014
Ethiopia	2011	India	2005–06
Gabon	2012	Nepal	2006, 2011
Ghana	2008, 2014	Timor-Leste	2009–10
Guinea	2012		
Kenya	2008–09, 2014	**European Region**	
Lesotho	2009, 2014	Azerbaijan	2006
Liberia	2007, 2013		
Madagascar	2008–09	**Eastern Mediterranean Region**	
Malawi	2010	Pakistan	2012–13
Mali	2012–13	Yemen	2013
Mozambique	2011		
Namibia	2006–07, 2013	**Western Pacific Region**	
Niger	2012	Cambodia	2005, 2010, 2014
Nigeria	2008, 2013		
Republic of the Congo	2011–12		
Rwanda	2010–11, 2014–15		
Sao Tome and Principe	2008–09		
Senegal	2010–11, 2012–15		
Sierra Leone	2008, 2013		
Tanzania	2010, 2015–16		
Togo	2013–14		
Uganda	2006, 2011		
Zambia	2007, 2013–14		
Zimbabwe	2010–11		

Study countries were selected based upon availability of DHS data, and are currently recommended by WHO to receive preventive chemotherapy.

Note, region classification is based upon World Health Organization definition.

**Table 2:** Characteristics of pre-school age children stratified by receipt of deworming, unmatched and CEM matched groups.

*Unmatched*				
Variable	Not dewormed	Dewormed	SD	Standardized mean difference
Age (years)	2.4	2.5	1.1	0.12
Gender (% female)	49.2	49	50	0
Wealth quintile	2.7	2.9	1.4	0.14
Mother’s age (% >30 years)	42.9	45.1	49.6	0.05
Rural (%)	70.4	65.8	46.5	0.1
Mother’s education tertile	0.9	1	0.8	0.21
Drinking water (% improved)	55.9	58.7	49.5	0.06
Toilet facility (% improved)	39.9	46.7	49.5	0.14
Receipt of 3^rd^ dose of DPT vaccine (% received)	61.7	81.3	44.7	0.44
*Matched (CEM)*				
Variable	Not dewormed	Dewormed	SD	Standardized mean difference
Age (years)	2.5	2.5	1.1	0
Gender (% female)	48.9	48.9	50	0
Wealth quintile	2.8	2.8	1.4	0
Mother’s age (% >30 years)	44.3	44.3	49.7	0
Rural (%)	67.6	67.6	46.8	0
Mother’s education tertile	1	1	0.8	0
Drinking water (% improved)	59.8	59.8	49	0
Toilet facility (% improved)	46	46	49.8	0
Receipt of 3^rd^ dose of DPT vaccine (% received)	82.9	82.9	37.7	0

CEM, coarsened exact matching; DPT, diphtheria-pertussis-tetanus

### Deworming and health outcomes

Globally, in the matched analysis, children who received deworming treatment were less likely to be stunted (1.2 percentage decline from mean of 36%; 95%CI [-1.9, -0.5%]; OR:0.92; p<0.001). This association was robust and mostly consistent, meaning this relationship was not observed in the negative-exposure controls, relaxing concern for any residual confounding (Table 3, A4, A5). We also found that children who received deworming treatment were less likely to be anemic (1.4 percentage decline from mean of 53%; 95%CI [-2.2, -0.6%]; OR:0.93; p<0.001), but while this finding was robust across specifications, it was also observed in negative-exposure controls suggesting the possibility of residual confounding. We did not measure a robust or consistent relationship between deworming and being underweight in the overall analysis (0.7 percentage decline from mean of 29%; 95%CI [-1.7, 0.2%]; OR:0.94; p = 0.06), but did measure a robust and consistent relationship between deworming and higher weight in the sub-group of underweight children (0.02 standard deviation increase; 95%CI [0.01, 0.04]; p<0.01).

**Table 3:** Regression model estimating the relationship in health outcomes for pre-school age children reported to have received treatment for intestinal worms in past 6 months.

	Underweight, global N = 280,473		Underweight, sub-Saharan Africa N = 165,100	
	Odds ratio (95% CI)	P-value	Odds ratio (95% CI)	P-value
Deworming	0.94 (0.89, 1.00)	0.06	0.93 (0.87, 1.00)	0.06
	Stunting, global N = 284,486		Stunting, sub-Saharan Africa N = 168,778	
	Odds ratio (95% CI)	P-value	Odds ratio (95% CI)	P-value
Deworming	**0.92 (0.89, 0.96)**	**<0.001**	**0.94 (0.89, 0.99)**	**0.01**
	Anemia, global N = 176,887		Anemia, sub-Saharan Africa N = 98,098	
	Odds ratio (95% CI)	P-value	Odds ratio (95% CI)	P-value
Deworming	**0.93 (0.89, 0.97)**	**<0.001**	**0.91 (0.86, 0.96)**	**<0.001**

Primary model was a logistic multivariable regression with coarsened exact matching and country survey-level and year fixed effects. The adjusted model matched on person-level covariates: child’s age and gender, mother’s age and education (no education, some primary school, completion of primary school or higher), wealth (composite measure of household living standard), rural residence, and access to improved water, toilet facilities, and healthcare (measured through third dose of DPT vaccine). Coarsened exact matching models do not include covariates in regression model since they are pre-processed for balance.

In sub-Saharan Africa, in the matched analysis of 32 countries, we found that children who received deworming treatment were less likely to be stunted (1.1 percentage decline from mean of 36%; 95%CI [-2.1, -0.2%]; OR: 0.94; p = 0.01) and less likely to be anemic (1.8 percentage decline from mean of 58%; 95%CI [-2.8, -0.7%]; OR:0.91; p<0.001) (Table 3). These findings were robust across the majority of alternative statistical models and mostly consistent in overall negative-exposure controls (Table A4, A5). We did not detect consistent associations between deworming and reduced risk of being underweight in the overall analysis, but did find a relationship in the sub-group of underweight children (0.02 standard deviation increase; 95%CI [0.00, 0.05]; p = 0.03). To conceptualize the magnitude of these effect sizes, we estimated that expanding deworming to 100% coverage could be related to 694,100 (95%UI: 134,300–1,298,700) averted cases of stunting and 850,600 (95%UI: 325,300–1,326,000) averted cases of anemia in pre-school age children from study countries in sub-Saharan Africa that are not yet being dewormed due to low coverage (Figure A4; Table A9).

### Sensitivity and other analyses

The primary findings reported as robust were supported across the majority of alternative statistical models (Table A4). In some cases, a finding was not consistent because the negative-exposure control was related to the health outcome (e.g. “access to condoms” was associated with less anemia in the global analysis) (Table A5). Deworming was associated with more reports of recent cough and fever as outcomes (see Appendix), which suggests some unexplained relationship, but argues against selection bias of better health in the population that received deworming. When matching with addition of covariate for receipt of vitamin A, use of malaria net, and breastfeeding, the main study finding remained broadly consistent; we did not find evidence for interaction between deworming and vitamin A. We repeated the main analysis restricting to only one child per household (i.e. not including additional children in the household) and found our findings to be broadly consistent.

We tested for dose-response effects through stratification by STH disease burden and age and testing continuous interaction; we found increasing effect size at older ages for the relationship between deworming and reduced stunting but did not find any other consistent relations (Table A6-A7). We did not find any clear evidence for larger effect sizes in higher STH disease burden settings (Table A7). Notably, we did not find any relationships between deworming and better health outcomes using data from non-endemic settings (country list in Appendix), which increases our confidence in our findings since we would not expect deworming benefits in these regions (Table A8). There was a range of effect sizes in the country-specific analyses (Figure A1-3).

## Discussion

Drawing upon data from more than 320,000 pre-school age children in 45 countries, this empirical analysis provides evidence for a global-level association between deworming and reduced stunting, with additional evidence for reduced anemia in sub-Saharan Africa for pre-school age children. We do not find a consistent relationship between deworming and improved weight. While the evidence to support the benefit of mass deworming has recently been questioned [1–4], this study aims to bring new data and study design to better understand the relationship between deworming and health outcomes in the population of pre-school age children. We addressed potential limitations of past studies by examining across diverse settings for generalizability and increasing sample size and focusing on children who received treatment to improve statistical power. Importantly, while a mother’s recall of deworming may be imperfect, any recall bias is unlikely to be unique to the dewormed group so this would bias our overall study findings towards no association and would not be a threat to the validity of our findings. The study results from our global empirical analysis provide evidence to support the deworming of pre-school age children.

Pre-school age children remain a key population for STH burden, because of their potential for long-term health and educational consequences, contribution to ongoing transmission, and need for treatment outside of traditional school-based deworming [5, 21]. Over the past 10 years, treatment in this age group has scaled up across many low and middle-income countries [27]. WHO currently estimates that global deworming has increased substantially to achieve a coverage of 51% of pre-school age children worldwide, although these estimates are only from countries that report this data [25]. Overall, the WHO estimate corresponds well with our estimate that 43% of pre-school age children had received deworming, which lends validity to the proxy-reported deworming exposure. However, we noted substantial within-country heterogeneity for deworming coverage, implying that some regions are not being reached despite a moderately high overall deworming coverage.

In our study, we measured a modest reduced risk of stunting among pre-school age children with a 1.1% decline from 36% prevalence–although this modest association corresponds with an estimated 694,100 cases of stunting in pre-school age children in our study countries from sub-Saharan Africa. This association should be conceptualized as the “diluted” population-level impact. In reality, the benefit of deworming is likely greatest among those children with high parasite burden and is intuitively null among those who are not infected. Therefore, the population-level effect is relatively modest. While stunting is considered to be a chronic indicator of health and may not be expected to change within the relatively short 6-month timeframe of our deworming exposure, reported receipt of deworming is likely correlated with previous deworming so this exposure may represent sustained periodic deworming. Our finding that older children had an increased association for improved height supports this hypothesis that larger benefits may be observed in repeatedly treated children.

We measured a consistent relationship between deworming and reduced risk of anemia (1.8% lower absolute percentage) in sub-Saharan Africa, which corresponds with an estimated 850,600 anemic pre-school age children in our study countries that could benefit from deworming. This relationship was not observed in the global analysis, driven by a non-association in South American countries. The disease burden of hookworm is likely higher in sub-Saharan Africa meaning a higher burden of avertable anemia, where we measured a positive association, than in South America, where we did not [34, 35].

We did not find a robust association between deworming and a reduction in being underweight in the primary analysis, and the negative-exposure control analysis suggested the possibility for residual confounding. However, there was an association between deworming and lower risk of being underweight in children who were already underweight. This suggests the possibility that deworming may associated with weight gain in children who are already underweight, but that the association is diluted out when including all children. A recent systematic review and meta-analysis of school-based deworming programs found positive effects of deworming on weight [16], while other meta-analyses failed to detect a measurable effect, although these have often focused on older children [3, 4].

Our study findings differ from the conclusions of recent meta-analyses of randomized trials, which failed to detect measurable improvements to height, weight, or anemia from deworming at a population-level [3, 4]. Notably, these meta-analyses have found that deworming may improve weight among those who are known to be infected but not at a population-level, which may be explained by a dilution effect [3]. A key difference in our analysis was a focus on pre-school age children, and measurement of only treated individuals rather than an overall population to increase statistical power (since overall population would dilute a relationship by including children who were not treated). A principal feature of this study was its large sample size and adequate statistical power. We included 66 national surveys with 325,000 children to ensure we could detect possible benefit despite the dilution effect (including non-infected and lightly infected children not expected to benefit from treatment). The discrepancy between our study findings and those of previous studies may further be explained by effect heterogeneity that is driven by spatial distribution of disease, differences between worm species, and an important non-linear relationship between disease burden (based on community infection intensity) and health outcomes [17, 18]. Our results find substantial country heterogeneity in the associations between deworming and health outcomes, which further supports the complexities in measuring the impact of deworming and the importance of evaluating real world deworming across diverse settings and epidemiologic conditions. While our deworming treatment record is reported for the previous 6 months, these children appear more likely to have received deworming treatments repeatedly, as recommended by WHO. Therefore, repeated periodic deworming, which consistently reduces worm burden, is more likely to result in long-term health-related outcomes and may also include treatment with different drugs (e.g. albendazole vs mebendazole), multiple treatments, and drugs for other parasitic worms (e.g. praziquantel for *Schistosoma* spp). Notably, this survey question may not have included treatment given for other infections (e.g. lymphatic filariasis) that would also be effective against STH infections, and this would bias our findings towards no association. We did not find a relationship between increasing country-level STH burden and higher effect sizes (i.e., dose response), although the aggregated country-level nature of the STH burden estimate may obscure a potential relationship. We did not perform a sub-analysis for Asia alone because data were limited.

The findings of this analysis should be interpreted within the context of the study design and constraints of the data. While we controlled for many key differences between the dewormed and control group through the CEM process to adjust for observed differences and through survey fixed effects to adjust for unobserved heterogeneities, residual unobserved differences may not be accounted for in the model (e.g. women who reported deworming may also have different health seeking behavior). We addressed this potential residual confounding by testing negative control exposures and outcomes. Negative-exposure controls that were associated with the health outcome raised concerns about selection bias that could not be eliminated with the observed data, even after matching; in these cases, the primary relationships were deemed inconsistent. While we measured an incompletely explained relationship between deworming and outcomes of increased frequency of reported fever and cough in the previous two weeks, this would bias against our primary study finding since these populations would not be predisposed to better health for unobserved reasons. Furthermore, we repeated the analysis with data from 10 non-endemic and low prevalence countries and found no consistently measurable associations between deworming and health outcomes, which may relax some concern for residual confounding since no relationship would be expected in these regions while similar confounding pathways may still be present. Similarly, while the main exposure variable for deworming of a child was proxy-reported by his or her mother and potentially subject to reporting bias (e.g. increased health knowledge) or recall bias, this would be more likely to bias our findings towards null unless the bias was unique and differential in the deworming group. Importantly, we did not have data on person-level STH infection status, although since mass deworming programs are also being implemented without knowledge of individual infection status, our goal was to quantify the population-level effectiveness of these real-world programs. The substantial overdispersion of the parasite and dilution effect of including all children (the majority uninfected or lightly infected) would significantly push our findings towards null. Since unprogrammed receipt of albendazole is common (e.g. local pharmacy, healthcare facility), it is likely that reported deworming included both programmed (e.g. mass drug administration, child health days) and unprogrammed sources [36, 37]. We also tested including vitamin A receipt as a variable or effect modifier in the model for sensitivity analysis, since vitamin A is often delivered alongside albendazole or mebendazole to pre-school age children during child health days, although our primary analyses remained robust even when considering vitamin A. Our analysis focused on pre-school age children, but future work should also examine school-age children and the broader community that may play a critical role in ongoing transmission [21, 23]. Finally, the estimates for potentially avertable cases of stunting and anemia related to deworming have substantial uncertainty, but are provided for better conceptualization of the magnitude of the study estimates and are not meant to imply causality.

This study found that deworming pre-school age children against STH is related to global reductions in risk of stunting, and evidence for reduced anemia in sub-Saharan Africa. Given recent controversy on deworming against STH, we provide an observational study design grounded in individual-level survey data that overcomes challenges in data and generalizability of prior work, and supports the continued deworming of pre-school age children.

## Supporting information

S1 Checklist STROBE checklist. (DOC)

S1 Appendix Supplemental materials. (DOCX)

## References

[1] Hicks JH, Kremer M, Miguel E. (2015) The Case for Mass Treatment of Intestinal Helminths in Endemic Areas. PLoS Negl Trop Dis 9(10):e0004214 doi: 10.1371/journal.pntd.0004214 . 26492528 10.1371/journal.pntd.0004214 PMC4619642

[2] Montresor A, Addiss D, Albonico M, Ali SM, Ault SK, Gabrielli AF, et al (2015) Methodological Bias Can Lead the Cochrane Collaboration to Irrelevance in Public Health Decision-Making. PLoS Negl Trop Dis 9(10):e0004165 doi: 10.1371/journal.pntd.0004165 . 26492178 10.1371/journal.pntd.0004165 PMC4619606

[3] Taylor-Robinson DC, Maayan N, Soares-Weiser K, Donegan S, Garner P. (2015) Deworming drugs for soil-transmitted intestinal worms in children: effects on nutritional indicators, haemoglobin, and school performance. Cochrane Database Syst Rev 7:CD000371. doi: 10.1002/14651858.CD000371.pub6 . 26202783 10.1002/14651858.CD000371.pub6 PMC4523932

[4] Welch VA, Ghogomu E, Hossain A, Awasthi S, Bhutta ZA, Cumberbatch C, et al (2017) Mass deworming to improve developmental health and wellbeing of children in low-income and middle-income countries: a systematic review and network meta-analysis. Lancet Glob Health 5(1):e40–e50. doi: 10.1016/S2214-109X(16)30242-X . 27955788 10.1016/S2214-109X(16)30242-X

[5] WHO. (2006) Preventive chemotherapy in human helminthiasis Coordinated use of anthelminthic drugs in control interventions: a manual for health professionals and programme managers. Geneva: World Health Organization.

[6] Pullan RL, Smith JL, Jasrasaria R, Brooker SJ. (2014) Global numbers of infection and disease burden of soil transmitted helminth infections in 2010. Parasit Vectors 7:37 Epub 2014/01/23. doi: 10.1186/1756-3305-7-37 . 24447578 10.1186/1756-3305-7-37 PMC3905661

[7] Awasthi S, Peto R, Pande VK, Fletcher RH, Read S, Bundy DA. (2008) Effects of deworming on malnourished preschool children in India: an open-labelled, cluster-randomized trial. PLoS Negl Trop Dis 2(4):e223 doi: 10.1371/journal.pntd.0000223 . 18414647 10.1371/journal.pntd.0000223 PMC2291568

[8] Alderman H, Konde-Lule J, Sebuliba I, Bundy D, Hall A. (2006) Effect on weight gain of routinely giving albendazole to preschool children during child health days in Uganda: cluster randomised controlled trial. BMJ 333(7559):122 doi: 10.1136/bmj.38877.393530.7C . 16790460 10.1136/bmj.38877.393530.7C PMC1502184

[9] Aiken AM, Davey C, Hargreaves JR, Hayes RJ. (2015) Re-analysis of health and educational impacts of a school-based deworming programme in western Kenya: a pure replication. Int J Epidemiol 44(5):1572–80. doi: 10.1093/ije/dyv127 . 26203169 10.1093/ije/dyv127 PMC4681107

[10] Miguel E, Kremer M. (2004) Worms: Identifying impacts on education and health in the presence of treatment externalities. Econometrica 72(1):159–217. doi: 10.1111/j.1468-0262.2004.00481.x PMID: WOS:000188202200006.

[11] Hall A, Hewitt G, Tuffrey V, de Silva N. (2008) A review and meta-analysis of the impact of intestinal worms on child growth and nutrition. Matern Child Nutr 4 Suppl 1:118–236. doi: 10.1111/j.1740-8709.2007.00127.x . 18289159 10.1111/j.1740-8709.2007.00127.x PMC6860651

[12] Smith JL, Brooker S. (2010) Impact of hookworm infection and deworming on anaemia in non-pregnant populations: a systematic review. Trop Med Int Health 15(7):776–95. doi: 10.1111/j.1365-3156.2010.02542.x . 20500563 10.1111/j.1365-3156.2010.02542.x PMC2916221

[13] Bethony J, Brooker S, Albonico M, Geiger SM, Loukas A, Diemert D, et al (2006) Soil-transmitted helminth infections: ascariasis, trichuriasis, and hookworm. Lancet 367(9521):1521–32. doi: 10.1016/S0140-6736(06)68653-4 . 16679166 10.1016/S0140-6736(06)68653-4

[14] Awasthi S, Peto R, Read S, Richards SM, Pande V, Bundy D, et al (2013) Population deworming every 6 months with albendazole in 1 million pre-school children in North India: DEVTA, a cluster-randomised trial. Lancet 381(9876):1478–86. doi: 10.1016/S0140-6736(12)62126-6 . 23498850 10.1016/S0140-6736(12)62126-6 PMC3647147

[15] Joseph SA, Casapia M, Montresor A, Rahme E, Ward BJ, Marquis GS, et al (2015) The Effect of Deworming on Growth in One-Year-Old Children Living in a Soil-Transmitted Helminth-Endemic Area of Peru: A Randomized Controlled Trial. PLoS Negl Trop Dis 9(10):e0004020 doi: 10.1371/journal.pntd.0004020 . 26426270 10.1371/journal.pntd.0004020 PMC4591279

[16] Croke K, Hicks JH, Hsu E, Kremer M, Miguel E. (2016) Does Mass Deworming Affect Child Nutrition? Meta-analysis, Cost-Effectiveness, and Statistical Power. NBER.

[17] Andrews JR, Bogoch II, Utzinger J. (2017) The benefits of mass deworming on health outcomes: new evidence synthesis, the debate persists. Lancet Glob Health 5(1):e4–e5. doi: 10.1016/S2214-109X(16)30333-3 . 27955787 10.1016/S2214-109X(16)30333-3

[18] Lo NC, Addiss DG, Hotez PJ, King CH, Stothard JR, Evans DS, et al (2016) A call to strengthen the global strategy against schistosomiasis and soil-transmitted helminthiasis: the time is now. Lancet Infect Dis. doi: 10.1016/S1473-3099(16)30535-7 . 27914852 10.1016/S1473-3099(16)30535-7 PMC5280090

[19] (2016) WHO weekly epidemiological record 49/50(91):585–600.

[20] Anderson R, Truscott J, Hollingsworth TD. (2014) The coverage and frequency of mass drug administration required to eliminate persistent transmission of soil-transmitted helminths. Philos Trans R Soc Lond B Biol Sci 369(1645):20130435 Epub 2014/05/14. doi: 10.1098/rstb.2013.0435 . 24821921 10.1098/rstb.2013.0435 PMC4024228

[21] Lo NC, Bogoch II, Blackburn BG, Raso G, N'Goran EK, Coulibaly JT, et al (2015) Comparison of community-wide, integrated mass drug administration strategies for schistosomiasis and soil-transmitted helminthiasis: a cost-effectiveness modelling study. Lancet Glob Health 3(10):e629–38. doi: 10.1016/S2214-109X(15)00047-9 . 26385302 10.1016/S2214-109X(15)00047-9

[22] Lo NC, Lai YS, Karagiannis-Voules DA, Bogoch II, Coulibaly JT, Bendavid E, et al (2016) Assessment of global guidelines for preventive chemotherapy against schistosomiasis and soil-transmitted helminthiasis: a cost-effectiveness modelling study. Lancet Infect Dis. doi: 10.1016/S1473-3099(16)30073-1 . 27286968 10.1016/S1473-3099(16)30073-1

[23] Truscott JE, Hollingsworth TD, Brooker SJ, Anderson RM. (2014) Can chemotherapy alone eliminate the transmission of soil transmitted helminths? Parasit Vectors 7:266 Epub 2014/06/12. doi: 10.1186/1756-3305-7-266 . 24916278 10.1186/1756-3305-7-266 PMC4079919

[24] Baird S, Hicks JH, Kremer M, Miguel E. (2016) Worms at work: Long-run impacts of a child health investment. Q J Econ. 10.1093/qje/qjw022 PMC5094294 27818531

[25] WHO. (2017) Weekly epidemiological record: Schistosomiasis and soil-transmitted helminthiases: number of people treated in 2016. 29218962

[26] Demographic and Health Surveys (DHS) 2005–2016. Available from: http://www.dhsprogram.com/Data/.

[27] World Health Organization PCT databank: Soil-transmitted helminthiases Geneva: World Health Organization.

[28] WHO. The WHO Child Growth Standards Geneva: World Health Organization.

[29] Bennett A, Eisele T, Keating J, Yukich J. (2015) Global Trends in Care Seeking and Access to Diagnosis and Treatment of Childhood Illnesses. Demographic and Health Surveys Working Papers

[30] WHO/UNICEF Joint Monitoring Programme (JMP) for Water Supply and Sanitation Available from: http://www.wssinfo.org/definitions-methods/watsan-categories/. 10.3390/ijerph17176262 PMC7503684 32872130

[31] Iacus SM, King G, Porro G. (2009) cem: Software for Coarsened Exact Matching. J Stat Softw 30(9).

[32] Vable AM, Kawachi I, Canning D, Glymour MM, Jimenez MP, Subramanian SV. (2016) Are There Spillover Effects from the GI Bill? The Mental Health of Wives of Korean War Veterans. PLoS One 11(5):e0154203 doi: 10.1371/journal.pone.0154203 . 27186983 10.1371/journal.pone.0154203 PMC4871362

[33] King G, Nielsen R, Coberley C, Pope JE. (2011) Comparative Effectiveness of Matching Methods for Causal Inference Working paper Harvard University, Institute for Quantative Social Science.

[34] Chammartin F, Scholte RG, Guimaraes LH, Tanner M, Utzinger J, Vounatsou P. (2013) Soil-transmitted helminth infection in South America: a systematic review and geostatistical meta-analysis. Lancet Infect Dis 13(6):507–18. doi: 10.1016/S1473-3099(13)70071-9 . 23562238 10.1016/S1473-3099(13)70071-9

[35] Karagiannis-Voules DA, Biedermann P, Ekpo UF, Garba A, Langer E, Mathieu E, et al (2015) Spatial and temporal distribution of soil-transmitted helminth infection in sub-Saharan Africa: a systematic review and geostatistical meta-analysis. Lancet Infect Dis 15(1):74–84. doi: 10.1016/S1473-3099(14)71004-7 . 25486852 10.1016/S1473-3099(14)71004-7

[36] Addiss DG. (2015) The challenge of unreported and unprogrammed deworming for soil-transmitted helminth control programs. Int Health 7(6):377–9. doi: 10.1093/inthealth/ihv055 . 26311691 10.1093/inthealth/ihv055

[37] Harris JR, Worrell CM, Davis SM, Odero K, Mogeni OD, Deming MS, et al (2015) Unprogrammed deworming in the Kibera slum, Nairobi: implications for control of soil-transmitted helminthiases. PLoS Negl Trop Dis 9(3):e0003590 doi: 10.1371/journal.pntd.0003590 . 25763577 10.1371/journal.pntd.0003590 PMC4357447

