# Supplementary material for: Deworming in pre-school age children: A global empirical analysis of health outcomes
Source: PLoS Negl Trop Dis. 2018 May 31;12(5):e0006500. doi: 10.1371/journal.pntd.0006500 (PMC5997348; doi:10.1371/journal.pntd.0006500)
Supplement: S1 Appendix — (DOCX) [file pntd.0006500.s002.docx]

**Supplemental Materials**

Lo NC, Snyder J, Addiss DG, Heft-Neal S, Andrews JR, and Bendavid E. Deworming in pre-school age children: a global empirical analysis of health outcomes. PLoS Negl Trop Dis (2018).

**Contents**

Section 1: Technical appendix.……………..…………….……….……..………………….Page 3

Section 2: Supplemental figures and tables…………………..…………….………….....…Page 6

**Section 1: Technical appendix**

In this technical appendix, we provide further information on the Demographic and Health Surveys (DHS), data processing, and statistical design.

*Main analysis*

We pre-processed our sample to improve balance between the treated and untreated groups using coarsened exact matching (CEM).^1-5^ The CEM method balanced the dewormed and untreated groups of pre-school age children by matching on key observed characteristics between the groups that were “coarsened” to a specified level.^1,2^ We implemented CEM by balancing on the person-level covariates listed in the Methods, which reduced imbalance on observable features of the study population by removing or weighting study observations to create two matched groups.

We estimated the potential impact of providing deworming in sub-Saharan Africa by computing the number of avertable cases of disease states that could be related to deworming. First, we identified the number of children (ages 1-4 years) in each country based on demographic data from the World DataBank and United Nations 2015 Revision of World Population Prospects. We then estimated the number that were underweight, stunted, or anemic based on country-specific epidemiologic estimates of the prevalence of these conditions.^6,7^ We applied the effect sizes estimated in our primary analysis, adjusting for country-specific prevalences of stunting, anemia, and being underweight; this represented the number of children in whom the disease state (e.g. stunting) could be possibly averted in relation to deworming based on the STH burden in our sample population.^6,7^ We subtracted the cases that would already be potentially averted based on current deworming (using current global coverage in pre-school age children), and then computed excess avertable cases relative to 100% (primary analysis) or 75% deworming coverage. We computed a 95% uncertainty interval (UI) based on the 95% confidence interval of the effect sizes of deworming on health outcomes. These estimates of potentially avertable cases related to deworming are provided for better conceptualization of the magnitude of the study estimates, and are not meant to imply causality.

*Sensitivity analysis*

We tested alternative statistical specifications, including: a) an ordinary least squares regression using continuous health outcomes for weight-for-age, height-for-age, and hemoglobin; b) adjusted logistic models without matching; c) regression models with country- or sub-national (region-level) fixed effects to control for time-invariant differences at varying geographic levels such as some environmental factors. We performed these alternative specifications in all combinations. We defined relationship robustness in reference to these alternative statistical models. We tested country-specific analyses and other possible covariates including receipt of vitamin A (which may act as a proxy for child health days, where both albendazole and vitamin A are given concurrently), use of a malaria net, and breastfeeding status. For each additional covariate, we repeated the primary analysis with the covariate included in CEM and regression model. We additionally tested interaction between binary receipt of vitamin A and deworming.

We conducted analyses stratified by a measure of estimated STH disease burden, since the theoretical benefit of deworming may be larger in higher burden settings (where infection intensity is higher). For this, we used the proportion of preschool-age children in a country estimated by WHO to be living in a region where STH infection prevalence is >20%, and for whom periodic deworming is therefore recommended.^8^ We also stratified by the child’s age since older children may have more cumulative STH exposure that would benefit from treatment or may be in a different phase of growth. The stratified analyses were repeated by using continuous interaction terms between deworming and STH burden or age.

Finally, we re-ran our analyses using negative controls for exposure and outcomes to measure the possibility of unobserved confounding (see explanation in Methods).^9-11^ We tested the model with pre-s negative exposure control of “heard of family planning on radio” and “access to condoms”, and negative outcome controls of “cough in last two weeks” and “fever in last two weeks.” These variables were pre-specified and selected based on prior usage as negative controls, and since they are less likely to be in the causal pathway between deworming and the outcomes of interest, but may share similar sources of bias (e.g., healthcare access or healthcare seeking behavior, health status, poor nutrition, etc.).^11^ We defined a relationship as consistent when the negative controls did not hold comparable associations to primary study findings. We also repeated the analysis on DHS surveys from non-endemic and low burden countries.

*Missing data*

We included children with complete survey record for health outcome, deworming status, and relevant covariates. Observations that were missing or marked as “don’t know,” “not reported,” etc. were dropped. These responses were often reported as 95, 98, 99, or “.”. The model covariates specified in the main analysis were missing in ~8% of the total sample, and were dropped. The following DHS surveys were excluded for the stated reasons:

| **Country** | **Survey years** | **Comments** |
| --- | --- | --- |
| Bangladesh | 2007 | No deworming exposure data. |
| Benin | 2006 | No deworming exposure data. |
| Colombia | 2010 | No deworming exposure data. |
| Mali | 2006 | No deworming exposure data. |
| Niger | 2006 | No deworming exposure data. |
| Rep. of the Congo | 2005 | No deworming exposure data. |
| Zimbabwe | 2005-06 | No deworming exposure data. |
| Indonesia | 2007, 2012 | No health outcomes data. |
| Pakistan | 2006-07 | No health outcomes data. |
| Philippines | 2008, 2013 | No health outcomes data. |
| Bolivia | 2008 | No data on access to water |
| Albania | 2008-09 | Not endemic for STH. |
| Egypt | 2005, 2008, 2014 | Not endemic for STH. |
| Jordan | 2007, 2012 | Not endemic for STH. |
| Maldives | 2009 | Not endemic for STH. |
| Ukraine | 2007 | Not endemic for STH. |
| Armenia | 2010 | Low burden; PCT recommended in minority (<10%) of children. |
| Gambia | 2013 | Low burden; PCT recommended in minority (<10%) of children. |
| Kyrgyz Republic | 2012 | Low burden; PCT recommended in minority (<10%) of children. |
| Swaziland | 2006-07 | Low burden; PCT recommended in minority (<10%) of children. |
| Tajikistan | 2012 | Low burden; PCT recommended in minority (<10%) of children. |
|  |  |  |

STH, soil-transmitted helminthiasis; PCT, preventive chemotherapy

*Analytic code*

We used Stata MP version 14 (StataCorp, College Station, Texas). The authors fully support the importance of data sharing and transparency in research, and analytic code are available on request to the corresponding author. All data files are available from the DHS database online.

**References**

1. Iacus SM, King G, Porro G. cem: Software for Coarsened Exact Matching. *J Stat Softw* 2009; **30**.

2. Iacus SM, King G, Porro G. Causal Inference without Balance Checking: Coarsened Exact Matching. *Polit Anal* 2011.

3. Vable AM, Kawachi I, Canning D, Glymour MM, Jimenez MP, Subramanian SV. Are There Spillover Effects from the GI Bill? The Mental Health of Wives of Korean War Veterans. *PLoS One* 2016; **11**: e0154203.

4. King G, Nielsen R, Coberley C, Pope JE. Comparative Effectiveness of Matching Methods for Causal Inference. *Working paper Harvard University, Institute for Quantative Social Science* 2011.

5. King G, Nielsen R. Why Propensity Scores Should Not Be Used for Matching. *Working paper Harvard University, Institute for Quantative Social Science* 2016.

6. Disease GBD, Injury I, Prevalence C. Global, regional, and national incidence, prevalence, and years lived with disability for 310 diseases and injuries, 1990-2015: a systematic analysis for the Global Burden of Disease Study 2015. *Lancet* 2016; **388**: 1545-602.

7. Stevens GA, Finucane MM, Paciorek CJ, et al. Trends in mild, moderate, and severe stunting and underweight, and progress towards MDG 1 in 141 developing countries: a systematic analysis of population representative data. *Lancet* 2012; **380**: 824-34.

8. World Health Organization PCT databank: Soil-transmitted helminthiases Geneva: World Health Organization.

9. Lipsitch M, Tchetgen Tchetgen E, Cohen T. Negative controls: a tool for detecting confounding and bias in observational studies. *Epidemiology* 2010; **21**: 383-8.

10. Arnold BF, Ercumen A, Benjamin-Chung J, Colford JM, Jr. Brief Report: Negative Controls to Detect Selection Bias and Measurement Bias in Epidemiologic Studies. *Epidemiology* 2016; **27**: 637-41.

11. Patil SR, Arnold BF, Salvatore AL, et al. The effect of India's total sanitation campaign on defecation behaviors and child health in rural Madhya Pradesh: a cluster randomized controlled trial. *PLoS Med* 2014; **11**: e1001709.

**Section 2: Supplemental Figures and Tables**

Figure A1: Country estimates for relationship between self-reported deworming and being underweight

Figure A2: Country estimates for relationship between self-reported deworming and stunting

Figure A3: Country estimates for relationship between self-reported deworming and anemia

Figure A4: Stunting and anemia cases potentially avertable in relation to deworming in sub-Saharan Africa

Table A1: Description of study country deworming and STH burden

Table A2: Study countries survey and health outcomes

Table A3: Regression model specifications

Table A4: Alternative regression models estimating the relationships in health outcomes for pre-school age children reported to have received treatment for intestinal worms

Table A5: Regression model testing negative exposure and outcome controls for relationship between self-reported deworming and health outcomes

Table A6: Regression model estimating the relationship between self-reported deworming and health outcomes stratified by age

Table A7: Regression model estimating the relationship between self-reported deworming and health outcomes stratified by STH disease burden

Table A8: Regression model between self-reported deworming and health outcomes in low burden and non-endemic settings

Table A9: Health outcomes avertable through deworming in sub-Saharan Africa with perfect coverage

Table A10: Health outcomes avertable through deworming in sub-Saharan Africa with 75% coverage


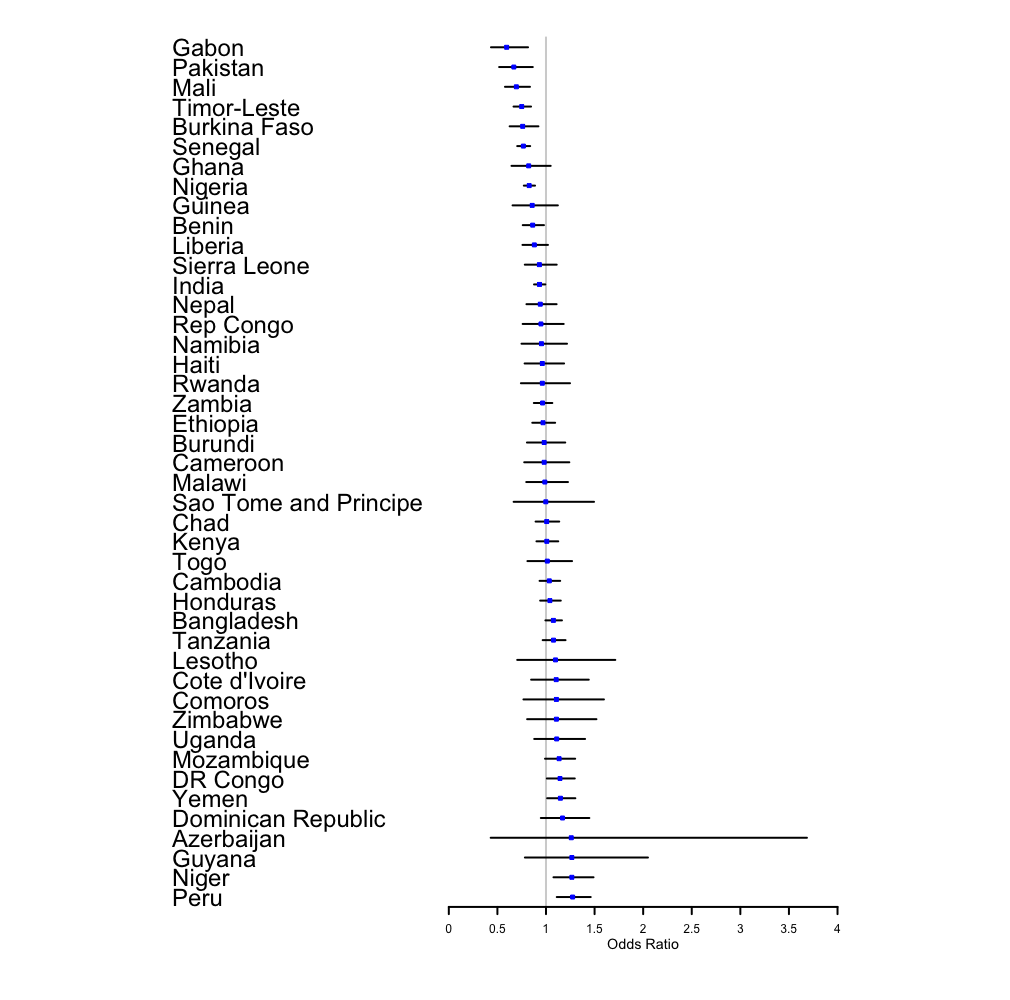


**Figure A1: Country estimates for relationship between self-reported deworming and being underweight**

**
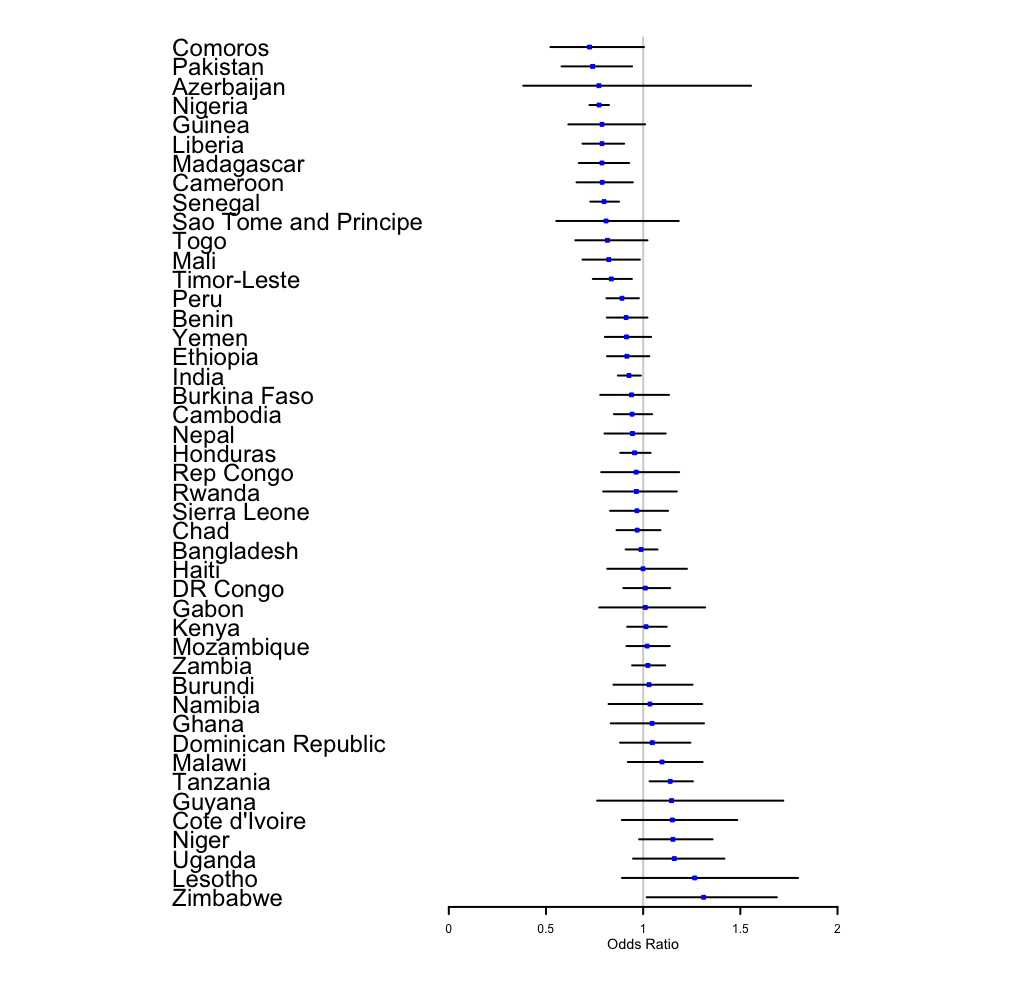
Figure A2: Country estimates for relationship between self-reported deworming and stunting**

**
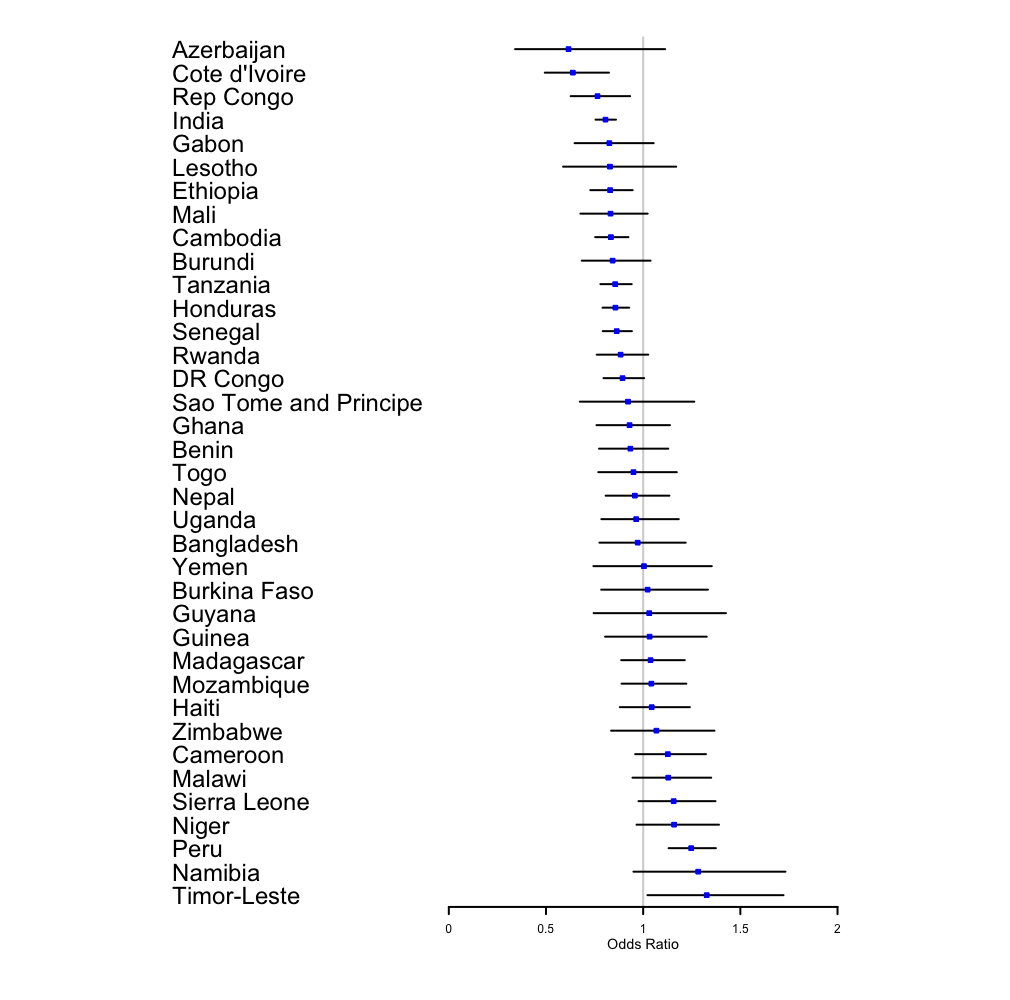
**

**Figure A3: Country estimates for relationship between self-reported deworming and anemia**

| **A** | 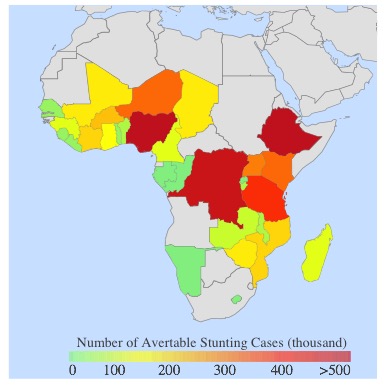 |
| --- | --- |
| **B** | 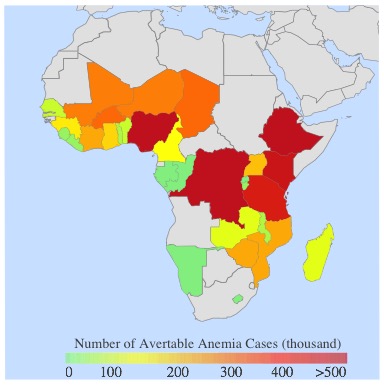 |

**Figure A4: Stunting and anemia cases potentially avertable in relation to deworming in sub-Saharan Africa.** We estimated the potential impact of expanding deworming in our study countries from sub-Saharan Africa in pre-school age children to estimate the number of potentially avertable cases of stunting (panel A) and anemia (panel B). We used the effect size from the primary analysis, country-specific prevalence of the stunting and anemia, and current deworming coverage for the country-level estimate. This map is meant to convey the magnitude of the primary study findings, and not meant to imply causality.

**Table A1: Description of study country deworming and STH burden**

| **Region** | **Study country** | **Deworming (%)** | **Children needing treatment (million)** |
| --- | --- | --- | --- |
|  |  |  |  |
| Africa | Benin | 50.6 | 1.6 |
|  | Burkina Faso | 14.2 | 7.2 |
|  | Burundi | 69.6 | 3.7 |
|  | Cameroon | 58 | 9 |
|  | Chad | 25.7 | 6 |
|  | Comoros | 59.6 | 0.2 |
|  | Côte d’Ivoire | 39.5 | 2.5 |
|  | Democratic Republic of the Congo | 60.7 | 26 |
|  | Ethiopia | 22.2 | 38.5 |
|  | Gabon | 77.8 | 0.6 |
|  | Ghana | 40.5 | 9.9 |
|  | Guinea | 31.2 | 2.1 |
|  | Kenya | 43.7 | 11.2 |
|  | Lesotho | 17.3 | 0.5 |
|  | Liberia | 57.2 | 1.8 |
|  | Madagascar | 75.2 | 9.6 |
|  | Malawi | 76.9 | 7.2 |
|  | Mali | 33.8 | 6.8 |
|  | Mozambique | 54.2 | 11.1 |
|  | Namibia | 30.5 | 0.8 |
|  | Niger | 29 | 9 |
|  | Nigeria | 21.6 | 49.1 |
|  | Republic of the Congo | 78.5 | 0.7 |
|  | Rwanda | 87.4 | 4.3 |
|  | Sao Tome and Principe | 59.6 | 0.08 |
|  | Senegal | 74.3 | 6.2 |
|  | Sierra Leone | 59.3 | 1.3 |
|  | Tanzania | 52.9 | 15.1 |
|  | Togo | 58 | 2.2 |
|  | Uganda | 50.7 | 15.6 |
|  | Zambia | 67 | 4.5 |
|  | Zimbabwe | 8.1 | 5.7 |
| Americas | Dominican Republic | 59.2 | 0.9 |
|  | Guyana | 58.7 | 0.2 |
|  | Haiti | 26.2 | 2.7 |
|  | Honduras | 60.5 | 2.4 |
|  | Peru | 33.4 | 2.8 |
| South-East Asia | Bangladesh | 51.2 | 47.5 |
|  | India | 16.5 | 223.1 |
|  | Nepal | 83 | 7.6 |
|  | Timor-Leste | 39.3 | 0.4 |
| European | Azerbaijan | 6.1 | 1.7 |
| Eastern Mediterranean | Pakistan | 24.5 | 31.1 |
|  | Yemen | 12.2 | 5.5 |
| Western Pacific | Cambodia | 50.9 | 5.6 |

**Table A2: Description of study country health outcomes**

| **Region** | **Study country** | **Underweight (%)** | **Stunting (%)** | **Anemia (%)** |
| --- | --- | --- | --- | --- |
|  |  |  |  |  |
| Africa | Benin | 29.4 | 45.1 | 57.4 |
|  | Burkina Faso | 32.8 | 34.2 | 87.6 |
|  | Burundi | 36.5 | 55.3 | 30.2 |
|  | Cameroon | 19.1 | 32.1 | 57.4 |
|  | Chad | 44.1 | 46.7 | -- |
|  | Comoros | 19.7 | 26.9 | -- |
|  | Côte d’Ivoire | 21.8 | 28.2 | 73.4 |
|  | Democratic Republic of the Congo | 32.5 | 45.4 | 60.2 |
|  | Ethiopia | 40.8 | 43.8 | 36.2 |
|  | Gabon | 12.4 | 22.5 | 62.3 |
|  | Ghana | 18.3 | 21.9 | 73.6 |
|  | Guinea | 26 | 31.6 | 74.8 |
|  | Kenya | 21.3 | 27.1 | -- |
|  | Lesotho | 18.1 | 35 | 30.2 |
|  | Liberia | 24.5 | 36.4 | -- |
|  | Madagascar | -- | 47.9 | 46.1 |
|  | Malawi | 19.1 | 45.6 | 58.8 |
|  | Mali | 33.8 | 37.8 | 80.1 |
|  | Mozambique | 18.8 | 37.6 | 63.9 |
|  | Namibia | 24.5 | 26.9 | 43.8 |
|  | Niger | 46 | 40.4 | 73.9 |
|  | Nigeria | 33.5 | 39.7 | -- |
|  | Republic of the Congo | 19.6 | 25.9 | 63.5 |
|  | Rwanda | 15.3 | 39.7 | 20.8 |
|  | Sao Tome and Principe | 19.9 | 23.4 | 57.4 |
|  | Senegal | 25.3 | 20.9 | 68.8 |
|  | Sierra Leone | 24.1 | 36.2 | 78.5 |
|  | Tanzania | 24.2 | 35.8 | 56.3 |
|  | Togo | 23 | 25.7 | 68.5 |
|  | Uganda | 21.3 | 34.5 | 53.6 |
|  | Zambia | 22 | 39.6 | -- |
|  | Zimbabwe | 16.3 | 29.5 | 46.2 |
| Americas | Dominican Republic | 5.2 | 8.1 | -- |
|  | Guyana | 12.6 | 18.1 | 35.7 |
|  | Haiti | 20 | 24 | 60.8 |
|  | Honduras | 13.9 | 27.9 | 28 |
|  | Peru | 7.6 | 21.9 | 21.1 |
| South-East Asia | Bangladesh | 46.7 | 36.8 | 49.4 |
|  | India | 47 | 43.2 | 62.4 |
|  | Nepal | 48.3 | 47.1 | 38.3 |
|  | Timor-Leste | 58.1 | 57.8 | 37.3 |
| European | Azerbaijan | 10.1 | 24.9 | 33.8 |
| Eastern Mediterranean | Pakistan | 35 | 45.3 | -- |
|  | Yemen | 48.5 | 46.3 | 80.8 |
| Western Pacific | Cambodia | 39 | 38.2 | 54.3 |

**Table A3: Regression model specifications**

| Variable | Category | Justification | Variable | Bin size |
| --- | --- | --- | --- | --- |
| Child’s age | Demographic/SES |  | Categorical | Year (ages 1-4) |
| Child’s gender | Demographic/SES |  | Binary | Male/female |
| Wealth | Demographic/SES |  | Continuous | Wealth quintile |
| Residence | Demographic/SES |  | Binary | Rural/urban |
| Mother’s age | Demographic/SES |  | Binary | Below 30 years; yes/no |
| Mother’s education | Demographic/SES |  | Continuous | 3-categories  (None, any up to primary, beyond primary) |
| Improved water access | Health |  | Binary | Yes/No |
| Improved toilet access | Health |  | Binary | Yes/No |
| Received 3^rd^ dose DPT vaccine | Health | Access to healthcare | Binary | Yes/No |
| Vitamin A in past 6 months | Health | Access to child health days | Binary | Yes/No |
| Country-level | Fixed effects |  | Categorical | Country |
| Time | Fixed effects |  | Categorical | Year |

SES; socioeconomic, DPT; diphtheria-pertussis-tetanus, STH; soil-transmitted helminthiasis

Covariates were chosen based upon literature from DHS surveys and health-seeking behavior and other potential sources of confounding.

**Table A4: Alternative regression models estimating the relationships in health outcomes for pre-school age children reported to have received treatment for intestinal worms**

| *Global* | Underweight | | Stunting | | Anemia | |
| --- | --- | --- | --- | --- | --- | --- |
| Model specification | Adjusted odds ratio (95% CI) | P-value | Adjusted odds ratio (95% CI) | P-value | Adjusted odds ratio (95% CI) | P-value |
| Multivariable logistic model  (survey fixed effects) | **0.95 (0.91, 0.99)** | **0.02** | **0.94 (0.91, 0.97)** | **<0.001** | **0.92 (0.88, 0.97)** | **0.001** |
| Multivariable logistic model  (region fixed effects) | **0.97 (0.95, 0.99)** | **0.02** | **0.95 (0.93, 0.97)** | **<0.001** | **0.93 (0.90, 0.95)** | **<0.001** |
| Matched logistic model  (region fixed effects) | **0.97 (0.94, 0.99)** | **0.02** | **0.94 (0.92, 0.97)** | **<0.001** | **0.92 (0.89, 0.96)** | **<0.001** |
| *Global* | Weight-for-age (z-score) | | Height-for-age (z-score ) | | Hemoglobin (g/L) | |
|  | Adjusted coeff (95% CI) | P-value | Adjusted coeff (95% CI) | P-value | Adjusted coeff (95% CI) | P-value |
| Multivariable OLS model  (survey fixed effects) | 0.01 (-0.02, 0.04) | 0.52 | **0.04 (0.01, 0.07)** | **0.009** | 0.49 (-0.05, 1.03) | 0.07 |
| Multivariable OLS model  (region fixed effects) | 0.00 (0.01, 0.01) | 0.66 | **0.03 (0.01, 0.04)** | **<0.001** | **0.43 (0.24, 0.62)** | **<0.001** |
| Matched OLS model  (survey fixed effects) | 0.00 (-0.02, 0.03) | 0.86 | **0.03 (0.01, 0.06)** | **0.02** | 0.34 (-0.09, 0.77) | 0.11 |
| Matched OLS model  (region fixed effects) | 0.01 (-0.01, 0.02) | 0.25 | **0.04 (0.02, 0.05)** | **<0.001** | **0.42 (0.16, 0.69)** | **0.002** |
| *Sub-Saharan Africa* | Underweight | | Stunting | | Anemia | |
| Model specification | Adjusted odds ratio (95% CI) | P-value | Adjusted odds ratio (95% CI) | P-value | Adjusted odds ratio (95% CI) | P-value |
| Multivariable logistic model  (survey fixed effects) | **0.91 (0.87, 0.96)** | **0.001** | **0.92 (0.88, 0.96)** | **<0.001** | **0.93 (0.89, 0.97)** | **0.001** |
| Multivariable logistic model  (region fixed effects) | **0.93 (0.91, 0.96)** | **<0.001** | **0.93 (0.91, 0.96)** | **<0.001** | **0.95 (0.92, 0.98)** | **0.004** |
| Matched logistic model  (region fixed effects) | **0.94 (0.91, 0.98)** | **0.001** | **0.94 (0.92, 0.97)** | **<0.001** | **0.94 (0.89, 0.98)** | **0.01** |
| *Sub-Saharan Africa* | Weight-for-age (z-score) | | Height-for-age (z-score ) | | Hemoglobin (g/L) | |
|  | Adjusted coeff (95% CI) | P-value | Adjusted coeff (95% CI) | P-value | Adjusted coeff (95% CI) | P-value |
| Multivariable OLS model  (survey fixed effects) | **0.04 (0.01, 0.07)** | **0.01** | **0.06 (0.02, 0.10)** | **0.004** | **0.55 (0.20, 0.90)** | **0.003** |
| Multivariable OLS model  (region fixed effects) | **0.03 (0.01, 0.04)** | **0.002** | **0.04 (0.02, 0.06)** | **<0.001** | **0.36 (0.10, 0.62)** | **0.006** |
| Matched OLS model  (survey fixed effects) | 0.02 (-0.01, 0.04) | 0.27 | 0.04 (-0.01, 0.08) | 0.09 | **0.56 (0.20, 0.92)** | **0.003** |
| Matched OLS model  (region fixed effects) | 0.01 (-0.00, 0.03) | 0.16 | **0.03 (0.01, 0.05)** | **0.003** | **0.43 (0.08, 0.77)** | **0.02** |

In multivariate models, we include the covariates outlined in the Methods section. Many covariates (e.g. wealth, age) have consistent relations with the health outcomes. We also tested the addition of alternative covariates including vitamin A, use of a malaria net, and breastfeeding status; these covariates did not change the overall conclusion. Although notably, for the analysis of relation between deworming and anemia in sub-Saharan Africa, the additional covariate of malaria net resulted in an estimate consistent with the primary analysis but of borderline statistical significance (OR: 0.96 [95% CI: 0.91, 1.00], p=0.07), although this is likely related to reduction in sample size from missing data in the malaria net variable. The other analyses remained consistent with the primary study findings.

**Table A5: Regression model testing negative exposure and outcome controls for relationship between self-reported deworming and health outcomes**

| *Global analysis* | | | | | |
| --- | --- | --- | --- | --- | --- |
| Negative control for exposure | Health outcome | Logistic, adjusted odds ratio (95% CI) | P-value | OLS, adjusted coeff (95% CI) | P-value |
| Heard of family planning* | Underweight | **0.95 (0.91, 0.99)** | **0.03*** | 0.01 (-0.01, 0.04) | 0.25* |
| Access to condom* | Underweight | **0.96 (0.91, 0.99)** | **0.04*** | **0.02 (0.00, 0.05)** | **0.04*** |
| Heard of family planning | Stunting | 0.97 (0.93, 1.00) | 0.07 | 0.02 (-0.01, 0.04) | 0.16 |
| Access to condom | Stunting | **0.95 (0.92, 0.99)** | **0.007** | 0.02 (-0.00, 0.04) | 0.10 |
| Heard of family planning | Anemia | **0.95 (0.91, 0.98)** | **0.006** | **0.51 (0.22, 0.80)** | **0.001** |
| Access to condom | Anemia | 0.97 (0.91, 1.04) | 0.44 | 0.25 (-0.25, 0.75) | 0.32 |
| Negative control for outcome | Exposure | Logistic, adjusted odds ratio (95% CI) | P-value |  |  |
| Had cough in last two weeks | Deworming | **1.20 (1.13, 1.26)** | **<0.001** |  |  |
| Had fever in last two weeks | Deworming | **1.17 (1.11, 1.25)** | **<0.001** |  |  |
| *Sub-Saharan Africa analysis* | | | | | |
| Negative control for exposure | Health outcome | Logistic, adjusted odds ratio (95% CI) | P-value | OLS, adjusted coeff (95% CI) | P-value |
| Heard of family planning | Underweight | 0.95 (0.88, 1.02) | 0.13 | 0.02 (-0.02, 0.06) | 0.34 |
| Access to condom | Underweight | **0.93 (0.87, 0.99)** | **0.02** | 0.03 (-0.00, 0.06) | 0.08 |
| Heard of family planning | Stunting | **0.96 (0.94, 0.99)** | **0.01** | 0.02 (-0.01, 0.05) | 0.18 |
| Access to condom | Stunting | **0.95 (0.91, 0.99)** | **0.04** | 0.02 (-0.01, 0.05) | 0.25 |
| Heard of family planning | Anemia | 0.96 (0.90, 1.01) | 0.12 | **0.52 (0.09, 0.96)** | **0.02** |
| Access to condom | Anemia | 1.00 (0.91, 1.09) | 0.99 | 0.44 (-0.25, 1.13) | 0.20 |
| Negative control for outcome | Exposure | Logistic, adjusted odds ratio (95% CI) | P-value |  |  |
| Had cough in last two weeks | Deworming | **1.17 (1.10, 1.24)** | **<0.001** |  |  |
| Had fever in last two weeks | Deworming | **1.16 (1.08, 1.24)** | **<0.001** |  |  |

Statistical model in analysis was a logistic or ordinary least squares (OLS) multivariable regression with coarsened exact matching and country survey-level and year fixed effects.

**Table A6: Regression model estimating the relationship between self-reported deworming and health outcomes stratified by age**

| *Global analysis* | | | | | |
| --- | --- | --- | --- | --- | --- |
|  |  | Logistic model |  | OLS model |  |
| Outcome | Age (years) | Adjusted odds ratio (95% CI) | P-value | Adjusted coeff  (95% CI) | P-value |
| Underweight | 1 | **0.94 (0.89, 0.99)** | **0.03** | 0.02 (-0.02, 0.05) | 0.36 |
| Underweight | 2 | **0.92 (0.85, 0.99)** | **0.02** | 0.01 (-0.03, 0.04) | 0.66 |
| Underweight | 3 | 0.93 (0.86, 1.01) | 0.08 | 0.02 (-0.01, 0.06) | 0.18 |
| Underweight | 4 | 1.01 (0.93 1.11) | 0.73 | -0.01 (-0.03, 0.02) | 0.52 |
| Stunting | 1 | 0.98 (0.93, 1.02)** | 0.31 | 0.02 (-0.02, 0.06) | 0.28 |
| Stunting | 2 | 0.96 (0.91, 1.01)** | 0.08 | 0.04 (0.00, 0.08) | 0.04 |
| Stunting | 3 | **0.89 (0.84, 0.93)**** | **<0.001** | **0.06 (0.02, 0.10)** | **0.002** |
| Stunting | 4 | **0.91 (0.87, 0.96)**** | **<0.001** | 0.03 (-0.01, 0.06) | 0.10 |
| Anemia | 1 | **0.89 (0.85, 0.95)** | **<0.001** | **0.71 (0.21, 1.22)**** | **0.007** |
| Anemia | 2 | **0.94 (0.89, 1.00)** | **0.04** | 0.23 (-0.33, 0.80)** | 0.41 |
| Anemia | 3 | **0.90 (0.83, 0.98)** | **0.01** | 0.52 (-0.11, 1.16)** | 0.11 |
| Anemia | 4 | 1.00 (0.91, 1.08) | 0.91 | -0.18 (-0.88, 0.51)** | 0.60 |
| *Sub-Saharan Africa analysis* | | | | | |
|  |  | Logistic model |  | OLS model |  |
| Outcome | Age (years) | Adjusted odds ratio (95% CI) | P-value | Adjusted coeff  (95% CI) | P-value |
| Underweight | 1 | **0.91 (0.85, 0.97)** | **0.007** | 0.03 (-0.02, 0.08) | 0.20 |
| Underweight | 2 | 0.91 (0.82, 1.01) | 0.09 | 0.02 (-0.03, 0.06) | 0.47 |
| Underweight | 3 | 0.95 (0.88, 1.02) | 0.15 | 0.03 (-0.01, 0.07) | 0.12 |
| Underweight | 4 | 0.97 (0.85 1.09) | 0.58 | 0.01 (-0.03, 0.05) | 0.65 |
| Stunting | 1 | 0.95 (0.90, 1.01)***** | 0.12 | 0.04 (-0.02, 0.09) | 0.20 |
| Stunting | 2 | 0.98 (0.91, 1.06)***** | 0.65 | 0.03 (-0.03, 0.10) | 0.33 |
| Stunting | 3 | **0.90 (0.84, 0.96)*** | **0.002** | **0.06 (0.01, 0.11)** | **0.01** |
| Stunting | 4 | **0.92 (0.86, 0.98)*** | **0.01** | 0.03 (-0.02, 0.09) | 0.21 |
| Anemia | 1 | **0.92 (0.86, 0.97)** | **0.004** | **0.64 (0.15, 1.12)** | **0.01** |
| Anemia | 2 | 0.93 (0.85, 1.01) | 0.07 | 0.37 (-0.20, 0.94) | 0.19 |
| Anemia | 3 | **0.87 (0.78, 0.98)** | **0.03** | **0.90 (0.26, 1.53)** | **0.007** |
| Anemia | 4 | 1.00 (0.92, 1.08) | 0.92 | 0.11 (-0.40, 0.62) | 0.67 |

Statistical model in analysis was a logistic or ordinary least squares (OLS) multivariable regression with coarsened exact matching and country survey-level and year fixed effects. We also formally tested interaction between deworming and country-level STH burden (*p<0.10, **p<0.05).

**Table A7: Regression model estimating the relationship between self-reported deworming and health outcomes stratified by STH disease burden**

| *Global analysis* | | | | | |
| --- | --- | --- | --- | --- | --- |
|  |  | Logistic model |  | OLS model |  |
| Outcome | Proportion of children needing PCT (%) | Adjusted odds ratio (95% CI) | P-value | Adjusted coeff  (95% CI) | P-value |
| Underweight | <50% | 0.99 (0.88, 1.10) | 0.80 | -0.03 (-0.10, 0.04) | 0.40 |
| Underweight | 50-75% | 0.97 (0.90, 1.05) | 0.43 | 0.02 (-0.03, 0.06) | 0.43 |
| Underweight | >75% | 0.94 (0.87, 1.03) | 0.17 | 0.00 (-0.03, 0.03) | 0.86 |
| Stunting | <50% | 0.94 (0.88, 0.99) | 0.04 | 0.03 (-0.02, 0.07) | 0.21 |
| Stunting | 50-75% | 0.94 (0.87, 1.02) | 0.12 | 0.04 (-0.02, 0.10) | 0.20 |
| Stunting | >75% | **0.94 (0.90, 0.98)** | **<0.01** | 0.02 (-0.02, 0.07) | 0.27 |
| Anemia | <50% | 1.01 (0.83, 1.23) | 0.91 | -1.33 (-3.27, 0.61) | 0.15 |
| Anemia | 50-75% | **0.87 (0.82, 0.92)** | **<0.001** | **1.32 (0.75, 1.89)** | **<0.001** |
| Anemia | >75% | **0.95 (0.91, 1.00)** | **0.06** | 0.29 (-0.05, 0.63) | 0.10 |
| *Sub-Saharan Africa analysis* | | | | | |
|  |  | Logistic model |  | OLS model |  |
| Outcome | Proportion of children needing PCT (%) | Adjusted odds ratio (95% CI) | P-value | Adjusted coeff  (95% CI) | P-value |
| Underweight | <50% | 0.92 (0.85, 1.00) | 0.06 | **0.05 (0.01, 0.09)** | **0.04** |
| Underweight | 50-75% | 0.97 (0.90, 1.06) | 0.52 | 0.01 (-0.04, 0.06) | 0.67 |
| Underweight | >75% | 0.91 (0.82, 1.02) | 0.11 | 0.02 (-0.03, 0.06) | 0.46 |
| Stunting | <50% | 0.96 (0.88, 1.05) | 0.37 | 0.05 (-0.01, 0.10) | 0.08 |
| Stunting | 50-75% | 0.95 (0.86, 1.04) | 0.27 | 0.03 (-0.05, 0.10) | 0.46 |
| Stunting | >75% | **0.93 (0.87, 0.99)** | **0.02** | 0.04 (-0.02, 0.10) | 0.19 |
| Anemia | <50% | 0.87 (0.72, 1.06) | 0.17 | 0.31 (-1.35, 1.97) | 0.65 |
| Anemia | 50-75% | **0.90 (0.85, 0.94)** | **<0.001** | **0.96 (0.43, 1.50)** | **<0.01** |
| Anemia | >75% | 0.96 (0.90, 1.02) | 0.15 | 0.27 (-0.16, 0.69) | 0.20 |

Data used is from WHO PCT database on proportion of children needing PCT; we tested the hypothesis that associations between deworming and health outcomes would be larger in higher burden settings that require higher proportions of their child population to receive PCT. While this sub-analysis can be informative, the relation between STH burden and expected health benefit is likely complex: e.g., if STH burden is too high, then while disease is highly prevalent, deworming school-aged children alone is unlikely to reduce transmission enough to measure health benefit. From this standpoint, a moderate burden setting is likely to benefit most from deworming since this setting would have sufficient disease burden, but also would be responsive to deworming programs. We also formally tested interaction between deworming and country-level STH burden, but did not find any interaction (p<0.10).

**Table A8: Regression model between self-reported deworming and health outcomes in low burden and non-endemic settings**

|  | Logistic model | | OLS model | |
| --- | --- | --- | --- | --- |
| Outcome | Adjusted odds ratio (95% CI) | P-value | Adjusted coeff  (95% CI) | P-value |
| Underweight | 1.05 (0.99, 1.11) | 0.11 | -0.02 (-0.08, 0.04) | 0.38 |
| Stunting | 0.99 (0.90, 1.08) | 0.78 | -0.02 (-0.16, 0.12) | 0.77 |
| Anemia | **1.11 (1.01, 1.21)** | **0.02** | 0.08 (-0.19, 0.35) | 0.46 |

This regression repeated the main analysis using DHS data from only non-endemic or low burden settings, which provides a negative control since no observed relationship would be expected for deworming in these settings.

**Table A9: Health outcomes avertable through deworming in sub-Saharan Africa with perfect coverage**

|  | **Avertable cases** | | | | | |
| --- | --- | --- | --- | --- | --- | --- |
| **Study country** | **Stunting** | | | **Anemia** | | |
|  |  | | |  | | |
|  | Base case | Lower | Upper | Base case | Lower | Upper |
| Benin | 7331 | 1419 | 13716 | 8472 | 3239 | 13207 |
| Burkina Faso | 20730 | 4012 | 38785 | 30636 | 11714 | 47756 |
| Burundi | 7124 | 1379 | 13328 | 3962 | 1515 | 6176 |
| Cameroon | 11643 | 2253 | 21784 | 13450 | 5143 | 20966 |
| Chad | 15752 | 3049 | 29471 | 30628 | 11711 | 47743 |
| Comoros | 403 | 78 | 754 | 498 | 190 | 777 |
| Côte d’Ivoire | 17854 | 3456 | 33404 | 22969 | 8782 | 35804 |
| Dem Rep of the Congo | 48267 | 9342 | 90306 | 54838 | 20967 | 85482 |
| Ethiopia | 123058 | 23818 | 230237 | 109134 | 41728 | 170121 |
| Gabon | 233 | 45 | 435 | 504 | 193 | 785 |
| Ghana | 13339 | 2582 | 24957 | 18302 | 6998 | 28529 |
| Guinea | 11429 | 2212 | 21383 | 16346 | 6250 | 25480 |
| Kenya | 30353 | 5875 | 56790 | 48064 | 18378 | 74924 |
| Lesotho | 1976 | 382 | 3697 | 1844 | 705 | 2874 |
| Liberia | 2568 | 497 | 4805 | 3933 | 1504 | 6130 |
| Madagascar | 10028 | 1941 | 18762 | 10567 | 4040 | 16472 |
| Malawi | 7073 | 1369 | 13233 | 6340 | 2424 | 9882 |
| Mali | 16287 | 3152 | 30472 | 28300 | 10821 | 44114 |
| Mozambique | 19914 | 3854 | 37258 | 23104 | 8834 | 36015 |
| Namibia | 1338 | 259 | 2503 | 1979 | 757 | 3085 |
| Niger | 30552 | 5913 | 57162 | 29434 | 11254 | 45882 |
| Nigeria | 191725 | 37108 | 358711 | 264416 | 101100 | 412178 |
| Rep of the Congo | 903 | 175 | 1689 | 1776 | 679 | 2769 |
| Rwanda | 2182 | 422 | 4082 | 1853 | 709 | 2889 |
| Sao Tome and Principe | 73 | 14 | 136 | 106 | 41 | 166 |
| Senegal | 3674 | 711 | 6875 | 9017 | 3448 | 14057 |
| Sierra Leone | 3333 | 645 | 6235 | 4069 | 1556 | 6343 |
| Tanzania | 38582 | 7468 | 72186 | 45309 | 17324 | 70629 |
| Togo | 2932 | 568 | 5487 | 6386 | 2442 | 9954 |
| Uganda | 28693 | 5554 | 53684 | 20309 | 7765 | 31658 |
| Zambia | 9135 | 1768 | 17091 | 10197 | 3899 | 15895 |
| Zimbabwe | 15631 | 3025 | 29246 | 23905 | 9140 | 37263 |
| TOTAL | 694115 | 134345 | 1298664 | 850647 | 325250 | 1326005 |

This model computes avertable cases relative to 100% deworming coverage (see Methods).

**Table A10: Health outcomes avertable through deworming in sub-Saharan Africa with 75% coverage**

|  | **Avertable cases** | | | | | |
| --- | --- | --- | --- | --- | --- | --- |
| **Study country** | **Stunting** | | | **Anemia** | | |
|  |  | | |  | | |
|  | Base case | Lower | Upper | Base case | Lower | Upper |
| Benin | 3621 | 701 | 6775 | 4185 | 1600 | 6523 |
| Burkina Faso | 14690 | 2843 | 27484 | 21709 | 8301 | 33841 |
| Burundi | 1265 | 245 | 2367 | 704 | 269 | 1097 |
| Cameroon | 4713 | 912 | 8817 | 5444 | 2082 | 8486 |
| Chad | 10452 | 2023 | 19555 | 20322 | 7770 | 31679 |
| Comoros | 154 | 30 | 288 | 190 | 73 | 296 |
| Côte d’Ivoire | 10476 | 2028 | 19601 | 13478 | 5153 | 21009 |
| Dem Rep of the Congo | 17563 | 3399 | 32859 | 19954 | 7629 | 31104 |
| Ethiopia | 83515 | 16164 | 156254 | 74065 | 28319 | 115455 |
| Gabon | 0 | 0 | 0 | 0 | 0 | 0 |
| Ghana | 7734 | 1497 | 14471 | 10612 | 4058 | 16542 |
| Guinea | 7276 | 1408 | 13613 | 10406 | 3979 | 16221 |
| Kenya | 16875 | 3266 | 31572 | 26721 | 10217 | 41654 |
| Lesotho | 1379 | 267 | 2579 | 1286 | 492 | 2005 |
| Liberia | 1068 | 207 | 1998 | 1636 | 625 | 2550 |
| Madagascar | 0 | 0 | 0 | 0 | 0 | 0 |
| Malawi | 0 | 0 | 0 | 0 | 0 | 0 |
| Mali | 10136 | 1962 | 18965 | 17613 | 6734 | 27455 |
| Mozambique | 9044 | 1750 | 16921 | 10493 | 4012 | 16356 |
| Namibia | 856 | 166 | 1602 | 1267 | 484 | 1975 |
| Niger | 19794 | 3831 | 37035 | 19070 | 7291 | 29726 |
| Nigeria | 130588 | 25275 | 244326 | 180100 | 68862 | 280743 |
| Rep of the Congo | 0 | 0 | 0 | 0 | 0 | 0 |
| Rwanda | 0 | 0 | 0 | 0 | 0 | 0 |
| Sao Tome and Principe | 28 | 5 | 52 | 41 | 15 | 63 |
| Senegal | 100 | 19 | 187 | 246 | 94 | 383 |
| Sierra Leone | 1286 | 249 | 2405 | 1570 | 600 | 2447 |
| Tanzania | 18103 | 3504 | 33871 | 21260 | 8129 | 33140 |
| Togo | 1187 | 230 | 2221 | 2585 | 988 | 4029 |
| Uganda | 14143 | 2737 | 26461 | 10010 | 3827 | 15604 |
| Zambia | 2215 | 429 | 4143 | 2472 | 945 | 3853 |
| Zimbabwe | 11379 | 2202 | 21290 | 17402 | 6654 | 27126 |
| TOTAL | 399640 | 77349 | 747712 | 494841 | 189202 | 771362 |

This model computes avertable cases relative to 75% deworming coverage (see Methods).
